# Could axillary lymph node dissection be omitted in the mastectomy patient with tumor positive sentinel node?

**DOI:** 10.3389/fonc.2023.1181069

**Published:** 2023-06-22

**Authors:** Ji Young You, Eun Sook Lee, Siew Kuan Lim, Youngmee Kwon, So-Youn Jung

**Affiliations:** ^1^ Breast and Endocrine Division, Department of Surgery, Korea University Medical Center, Seoul, Republic of Korea; ^2^ Center for Breast Cancer, National Cancer Center, Research Institute and Hospital, Goyang, Gyeonggi, Republic of Korea; ^3^ Department of Surgery, Breast Service, Changi General Hospital, Singapore, Singapore

**Keywords:** sentinel lymph node biopsy, mastectomy, node positive breast cancer, ACOSOG Z0011, axilla dissection

## Abstract

**Background:**

Recent data from the ACOSOG Z0011 trial suggest that axillary lymph node dissection (ALND) may not be necessary for patients with positive sentinel lymph node biopsy (SLNB) receiving breast-conserving surgery (BCS) with irradiation. However, consensus statements and guidelines have recommended that patients undergoing mastectomy with tumor-positive sentinel node undergo completion ALND. In this study, we compared the locoregional recurrence rate of patients with tumor-positive sentinel nodes among three groups: mastectomy with SLNB, mastectomy with ALND and BCS with SLNB.

**Method:**

We identified 6,163 women with invasive breast cancer who underwent surgical resection at our institution between January 2000 and December 2011. Clinicopathologic data obtained from the prospectively collected medical database were analyzed retrospectively. Among the patients with sentinel node positive, mastectomy with SLNB was performed in 39 cases, mastectomy with ALND in 181 cases, and BCS with SLNB in 165 cases. The primary end point was the loco-regional recurrence rate.

**Results:**

Clinicopathologic characteristics were similar among the groups. There were no cases of loco-regional recurrence in the sentinel groups. At a median follow-up of 61.0 months (last follow-up May 2013), the loco-regional recurrence rate of each group was 0% for BCS with SLNB and mastectomy with SLNB only, and 1.7% for mastectomy with ALND (*p*=0.182).

**Conclusion:**

In our study, there was no significant difference in loco-regional recurrence rates between groups. This result lends weight to the argument that SLNB without ALND may be a reasonable management for selected patients with appropriate surgery and adjuvant systemic therapy.

## Background

In the early 1990s, introduction of the sentinel lymph node (SLN) concept revolutionized the axillary surgery in breast cancer (1). As a consequence, patients with a negative SLN can now avoid unnecessary axillary lymph node dissection (ALND) and its attendant morbidity (2). In 2001, an international multicenter phase III trial was initiated by the European Organization for Research and Treatment of Cancer (EORTC). The After Mapping of the Axilla: Radiotherapy of Surgery? (AMAROS) study compared ALND and axillary radiation therapy (ART) in patients with early breast cancer with tumor-positive sentinel nodes (3). Results from this study showed that axillary dissection had no influence on the administration of adjuvant treatment in the first 566 patients assessed. More recently, in 2004 the American College of Surgeons Oncology Groups (ACOSOG) started the Z0011 Trial, a multicenter randomized trial investigating loco-regional recurrence after sentinel lymph node biopsy (SLNB) with or without axillary dissection in patients with sentinel lymph node metastasis (4). This study enrolled patients with cancers 5 cm or smaller and 1-2 positive SLNs who were treated with breast-conserving surgery (BCS). These patients were randomized to receive ALND and breast radiation therapy (BRT) or BRT alone (5). The results were published in 2010, and the authors concluded that ALND was not necessary for all women with one or two positive axillary sentinel lymph nodes who undergo breast conserving surgery, adjuvant systemic therapy, and opposing tangential field whole breast irradiation (6).

Many breast oncologists consider that the findings of Z0011 shifted the surgical paradigm in breast cancer. The 2012 NCCN guidelines (7) now recommend considering no further surgery for clinically node-negative patients with tumors under 5 cm in size and one or two positive SLNs who will receive BRT (7, 8). Based on this evidence, a number of experts at the St. Gallen Consensus Conference in 2013 voted that axillary dissection can be omitted in patients with macrometastases in 1-2 sentinel nodes who undergo breast conserving surgery and planned radiotherapy. However, they did not agree that axillary dissection can safely be omitted in patients with macrometastases in 1-2 sentinel nodes who undergo mastectomy without planned radiotherapy (9).

Fisher previously argued that breast cancer is a systemic disease, and providing systemic therapy in accordance with the hypothesis is generating good results in breast cancer outcomes (10). For patients with axillary node metastases who undergo mastectomy, there is no definite proof that complete axillary dissection or radiotherapy is required as a local control. In this preliminary study, we compared patients with axillary node metastasis who underwent mastectomy and did not undergo ALND against patients who received BCS and irradiation with SLNB only and those who received mastectomy with ALND. We analyzed the locoregional recurrence rate and found no differences in outcomes among the three groups.

## Methods

**Figure 1:**
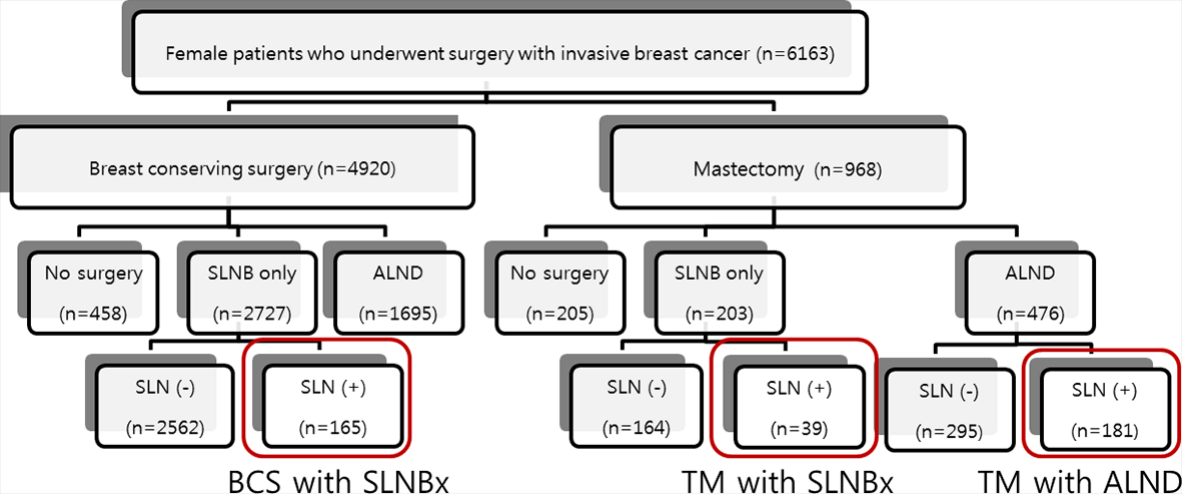
Study flow.

Our study is a retrospective study based on an existing prospective breast cancer database of the National Cancer Center, Goyang-si, Gyeonggi-do, Korea. We identified 6,163 consecutive women with invasive breast cancer who underwent surgery at our institution between January 2000 and December 2011. We excluded patients who had bilateral breast cancer or had no axillary surgery. All patients who were node-positive on sentinel biopsy were initially enrolled in this study. To observe the effects of different types of surgery on the breast and axilla, we compared the outcome of patients who underwent mastectomy with SLNB (n=39) only with that of patients who underwent BCS with SLNB (n=165) and those who underwent mastectomy with ALND (n=181). Patients who underwent BCS with ALND (n=104) were excluded from this study. The patient flow is outlined in **Figure 1**. We collected clinicopathologic information on these 385 patients by reviewing the prospective database of our institution for data on patient and tumor characteristics, breast surgery, sentinel-node biopsy, axillary lymph node dissection, radiotherapy, systemic adjuvant therapy, disease recurrence, and death during follow-up. The primary end point was the loco-regional recurrence rate.

Written informed consent was provided before surgery by all patients and this study was approved by the Institutional Review Board of our institution.

Patients were divided into three groups as above to perform statistical analysis. Associations among categorical variables were analyzed using chi-square test and one-way ANOVA. ER and PgR expression levels were divided into binary covariates based on Allred score (low score ≤6 and high score ≥7). Stepwise multivariate logistic regression analysis was performed to identify the significant factors associated with each surgery. Multivariate Cox regression analysis was used to compare recurrence-free survival according to surgery and to represent survival curves. A *p*-value less than or equal to 0.05 was considered to be statistically significant. All statistical analyses were performed with STATA Version 10 (StataCorp LP, College Station, TX, USA).

## Results

Of the 6,163 patients, 4,920 underwent breast-conserving surgery and 968 underwent mastectomy. **Table 1** lists basic patient, tumor, and treatment characteristics. In general, there were no significant differences in clinical and tumor characteristics among the three groups. The median ages were 47.0 years for the mastectomy with SLNB group, 48.0 years for the mastectomy with ALND group, and 46.0 years for the BCS group (*p*=0.373). The mean tumor size was 3.1 cm for the mastectomy with SLNB group, 3.0 cm for the mastectomy with ALND group, and 2.0 cm for the BCS group, representing a statistically significant difference (*p*=0.024). The number of patients treated with radiotherapy (RTx.) was significantly different among the groups (*p*<0.05). The extent of RTx. also differed; patients in the mastectomy group were more likely to have chest wall RTx. including supraclavicular nodes or internal mammary nodes, whereas most of the patients in the BCS group underwent breast radiation only (**Table 1**). Other than tumor size, T stage, and radiotherapy, other factors including lymphovascular invasion, hormonal receptor and HER2 receptor status, and tumor type, were not significantly different among the groups. The median number of nodes removed in the two SLNB groups was approximately 3 (3.5 in the mastectomy group and 3.1 in the BCS group).

**Table 1:** Clinicopathologic characteristics of the study population.

	BCS with SLNB (n=165)	TM with SLNB (n=39)	TM with ALND (n=181)	p-value
Age				0.373
Median	46.0	47.0	48.0	
Range	29-74	24-82	30-89	
Menopausal status				0.323
Premenopause	106(64.2%)	24(61.5%)	102(56.4%)	
Postmenopause	59(35.8%)	15(38.5%)	79(43.6%)	
Mean body mass index (kg/m^2^)	23.3738	24.1893	23.8877	0.552
T stage				0.000
T1	86(52.1%)	13(33.3%)	56(30.9%)	
T2	73(44.2%)	14(35.9%)	109(60.2%)	
T3	5(3.0%)	4(10.3%)	11(6.1%)	
Mean tumor size (cm)	2.0319	3.1205	3.0498	0.024
Tumor grade				0.033
Grade I	20(12.1%)	1(2.6%)	13(7.2%)	
Grade II	94(57.0%)	21(53.8%)	90(49.7%)	
Grade III	48(29.1%)	16(41.0%)	78(43.1%)	
Retrieved node number				0.000
Mean	3.15	3.59	14.55	
Range	01-Jun	01-May	Jun-38	
N stage				0.010
N1	162(98.2%)	34(87.2%)	177(97.8%)	
N2	3(1.8%)	4(10.3%)	2(1.1%)	
Lymphovascular invasion (LVI)				0.410
Present	141(85.5)	36(92.3%)	152(84.0%)	
Absent	24(14.5)	3(7.7%)	29(16.0%)	
Estrogen receptor (ER)				0.356
ER+	137(83.0%)	31(79.5%)	139(76.8%)	
ER-	28(17.0%)	8(20.5%)	42(23.2%)	
Progesterone receptor (PgR)				0.003
PgR+	135(81.8%)	30(76.9%)	119(65.7%)	
PgR-	30(18.2%)	9(23.1%)	62(34.3%)	
HER2 receptor				0.304
HER2+	32(19.4%)	11(28.2%)	46(25.4%)	
HER2-	133(80.6%)	28(71.8%)	135(74.6%)	
Tumor type				0.859
Invasive ductal	158(95.8%)	37(94.9%)	171(94.5%)	
Invasive lobular	7(4.2%)	2(5.1%)	10(5.5%)	
Adjuvant Therapy (Tx.)				
Chemotherapy (CTx.)	162(98.2%)	36(92.3%)	165(91.2%)	0.016
Neoadjuvant	3(1.9%)	3(8.3%)	6(3.6%)	
Endocrine therapy	140(84.8%)	32(82.1%)	144(79.6%)	0.501
Radiotherapy (RTx.)	165(100%)	20(51.3%)	90(49.7%)	<0.001
Breast only	131(79.4%)	4(20.0%)	16(17.8%)	
Extended	34(20.6%)	16(80.0%)	74(82.2%)	

BCS, breast-conserving surgery; TM, total mastectomy; SLNBx, sentinel lymph node biopsy; ALND, axillary lymph node dissection.

**Figure 2:**
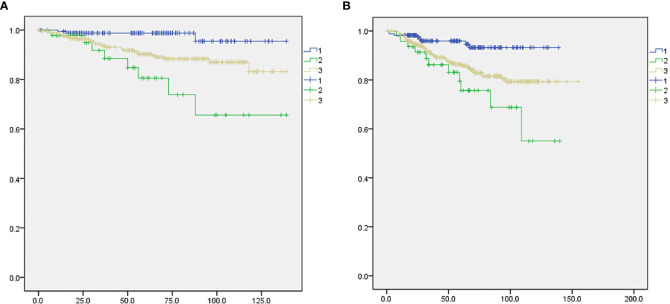
Comparison of disease-free survival and overall survival among the three study groups. 1: BCS with SLNB (blue), 2: mastectomy with SLNB (green), 3: mastectomy with ALND (gray). **(A)** Disease-free survival, *p*=0.018. **(B)** Overall survival, *p*=0.017.

At a median follow-up of 61.0 months, the loco-regional recurrence rates were 0% in the mastectomy with sentinel lymph node biopsy only group, 0% in the breast conserving surgery with SLN group and 1.7% (3 cases) in the mastectomy with axillary dissection group; however, the difference was not statistically significant (*p*-value=0.182). The total and systemic recurrence rates were higher in the mastectomy group than the breast-conserving group and this difference was statistically significant (*p* < 0.05). The length of disease-free survival and overall survival was not significantly different among the three groups (**Table 2**). However, using the log-rank test, the mastectomy with sentinel biopsy group showed a worse prognosis than the other two groups for both disease-free survival (**Figure 2A**, *p*=0.018) and overall survival (**Figure 2B**), *p*=0.017).

**Table 2:** Comparison of recurrence rate and survival.

	BCS with SLNBx. (n=165)	TM with SLNBx. (n=39)	TM with ALND (n=181)	p-value
Median follow up	57 months	59 months	63 months	0.077
Recurrence				
Total	3(1.8%)	6(15.4%)	9(5.0%)	0.001
Loco-regional	0	0	3(1.7%)	0.182
Systemic	2(1.2%)	6(15.4%)	6(3.3%)	0
DFS(months)	56	55	62	0.227
OS(months)	56	61	64	0.077

BCS, breast-conserving surgery; TM, total mastectomy; SLNBx, sentinel lymph node biopsy; ALND, axillary lymph node dissection; DFS, disease free survival; OS, overall survival.

**Table 3** shows the logistic regression analysis for loco-regional recurrence. Univariate analysis including all patients revealed that T stage (*p*=0.006, HR=6.506x10^-7^) and surgery type (*p*=0.026, HR=3.475 [1.159-10.419]) were statistically significant factors. However, in multivariate analysis neither factor was independently associated with recurrence (T stage: *p*=0.212, surgery: *p*=0.247).

**Table 3:** Logistic regression analysis for recurrence.

	Univariate			Multivariate		
	HR	95% CI of HR	p-value	HR	95% CI of HR	p-value
Age	0.953	0.905-1.004	0.069			
Menopause	1.757	0.613-5.033	0.294			
T stage	6.506x10^7^		0.006	3.309x10^7^		0.212
Tumor grade	0.679	0.262-1.754	0.424			
N stage	7.714x10^7^		0.607			
LVI	0.725	0.162-3.241	0.673			
ER	1.549	0.535-4.483	0.420			
PgR	1.432	0.523-3.920	0.485			
HER2	0.772	0.267-2.227	0.632			
Surgery	3.475	1.159-10.419	0.026	2.097	0.598-7.347	0.247
CTx.	0.000		0.998			
endoTx.	1.348	0.430-4.230	0.609			
RTx.	0.549	0.156-1.937	0.351			

*HR, hazard ratio; CI, confidence interval; LVI, Lymphovascular invasion; ER, estrogen receptor; PgR, progesterone receptor; Tx, Adjuvant Therapy; CTx, Chemotherapy; RTx, radiotherapy.

## Discussion

In the American College of Surgeons Oncology Group Z0011 randomized trial, ALND did not significantly affect overall survival or disease-free survival of patients with clinical T1-T2 breast cancer and a positive SLN who were treated with lumpectomy, adjuvant systemic therapy, and tangential-field whole breast radiation therapy (11, 12). No significant benefit in loco-regional control was seen with completion ALND despite the removal of additional tumor-involved lymph nodes (4). Yet despite increasing evidence that many women will not have additional nodal metastasis upon completion ALND, management of the patient with clinically negative, histologically positive lymph nodes undergoing mastectomy has not changed, and ALND remains the gold standard because of relatively insufficient evidence for mastectomy patients compared with BCS patients (13).

In this study, at a median follow-up of 61.0 months, we noted no difference among patients undergoing breast-conserving surgery with sentinel lymph node biopsy, mastectomy with axillary dissection, or mastectomy with sentinel lymph node biopsy groups with respect to the primary endpoint of loco-regional recurrence rate (panel). Although the number of enrolled patients was small, the protocol-specified criterion of non-inferiority of the mastectomy with sentinel lymph node biopsy only group was fulfilled. However, both disease-free survival and overall survival of the mastectomy with sentinel biopsy group were worse than the other two groups (**Figure 2**), possibly because of the higher initial tumor stage (**Table 1**). In addition, the radiotherapy rate in each group was different (**Table 1**). Due to the retrospective nature of this study, as well as the small number of case patients and limited data, we could not control for the rate and extent of radiotherapy.

Some previously published studies had a similar concept to ours. Crawford et al. (14) reported that routine completion axillary lymph node dissection for positive sentinel nodes in patients undergoing mastectomy was not associated with improved local control. They performed a retrospective review of women with stage 1 to 3 breast cancer and found that survival curves showed no significant difference in recurrence-free survival between the sentinel biopsy only group and axillary dissection group. Spiguel et al. (15) performed a retrospective analysis of a sentinel node-positive group staged as N1micro or N1. Their study included 123 node-positive patients who underwent SLNB alone with no completion axillary dissection for invasive breast cancer, among which approximately 30% of the patients underwent mastectomy. These patients showed a locoregional recurrence rate of 0.8%, and only one axillary recurrence, which is consistent with our results.

Several retrospective studies have reported low axillary recurrence rates in women with positive sentinel nodes who did not have completion ALND for various reasons (16–18). These retrospective studies are limited by their small numbers, limited knowledge of the reasons for not performing ALND, small number of sentinel node metastases, and lack of controls. Lannin et al. discussed some limitations of the results of ACOSOG Z0011 study in their essay (19). They showed that the Yale data confirmed the accuracy of the two Louisville models and reported that tumor size, number of positive sentinel nodes, and proportion of positive sentinel nodes were all significant predictors of prognosis. As differences in T stage or tumor size can be confounders, we compared groups of breast-conserving surgery with sentinel node biopsy, mastectomy with sentinel node biopsy, and mastectomy with axillary node dissection. We believe that comparing different surgery types reduces the fore-mentioned confounding effects.

Regardless of some limitations due to its retrospective nature, our study can be regarded as a preliminary study with sufficient value because we performed treatment with a relatively consistent policy at a single institution. The results of our study suggest that we can safely omit ALND in some patients with positive sentinel nodes who undergo mastectomy.

## Conclusion

Our study showed no difference in loco-regional recurrence rates among these three groups. Our results lend weight to the argument that SLNB without ALND may be reasonable management for selected patients who can be treated with appropriate surgery and adjuvant systemic therapy. Further validation through a well-designed prospective randomized clinical trial with a larger study population and using a standard protocol is needed.

## Data availability statement

The original contributions presented in the study are included in the article/**Supplementary Materials**, further inquiries can be directed to the corresponding author/s.

## Ethics statement

All procedures performed in studies involving human participants were in accordance with the ethical standards of the institutional and/or national research committee and with the 1964 Helsinki declaration and its later amendments or comparable ethical standards. The studies involving human participants were reviewed and approved by National Cancer Center. Written informed consent to participate in this study was provided by the patients (IRB No.NCC2016-0130)

## Author contributions

JY and EL conceived and designed the study. JY and SL wrote the paper. JY and S-YJ designed the figures. JY and YK summarized the data, analyzed the data and created the tables. EL, SL and S-YJ reviewed and edited the manuscript. All authors contributed to the article and approved the submitted version.
